# Predicting the bodily self in space and time

**DOI:** 10.1038/s41598-024-65607-y

**Published:** 2024-06-27

**Authors:** D. M. L. de Boer, P. J. Johnston, F. Namdar, G. Kerr, A. Cleeremans

**Affiliations:** 1 School of Exercise and Nutrition Sciences, Faculty of Health, Queensland University of Technology, 149 Victoria Park Road, Kelvin Grove, QLD 4059 Australia; 2 Consciousness, Cognition, and Computation Group (CO3), Centre for Research in Cognition and Neurosciences (CRCN), ULB Neuroscience Institute (UNI), Université Libre de Bruxelles (ULB), Avenue F.D. Roosevelt 50, CP191, 1050 Brussels, Belgium; 3 Information Sciences Division, Defence Science and Technology Group (DSTG), Eagle Farm, QLD 4009 Australia; 4 Design doc, Gerardt Burghoutweg 23, 1111 BW Diemen, The Netherlands

**Keywords:** Bodily self-consciousness, Embodied gaming, Full-body illusion (FBI), Prospective agency, Self-location, Social cognition, Virtual reality (VR), Consciousness, Agency, Cognitive control, Human behaviour

## Abstract

To understand how the human brain distinguishes itself from external stimulation, it was examined if motor predictions enable healthy adult volunteers to infer self-location and to distinguish their body from the environment (and other agents). By uniquely combining a VR-setup with full-body motion capture, a full-body illusion paradigm (FBI) was developed with different levels of motion control: (A) a standard, passive FBI in which they had no motion control; (B) an active FBI in which they made simple, voluntary movements; and (C) an immersive game in which they real-time controlled a human-sized avatar in third person. Systematic comparisons between measures revealed a causal relationship between (i) motion control (prospective agency), (ii) self-other identification, and (iii) the ability to locate oneself. Healthy adults could recognise their movements in a third-person avatar and psychologically align with it (action observation); but did not lose a sense of place (self-location), time (temporal binding), nor who they are (self/other). Instead, motor predictions enabled them to localise their body and to distinguish self from other. In the future, embodied games could target and strengthen the brain’s control networks in psychosis and neurodegeneration; real-time motion simulations could help advance neurorehabilitation techniques by fine-tuning and personalising therapeutic settings.

## Introduction

This study investigates a potentially critical link between self-action and embodiment, which may shed light on how the human brain evolved to be a conscious agent. We propose that this may have arisen out of a need for the brain to recognise and monitor self- versus other purposeful actions and their outcomes (i.e., prospective agency, see below). A crucial step for a brain to develop self-consciousness is to distinguish itself from the environment. This is related to the deeper question of how the brain separates the signals it generates itself (i.e., actions, thoughts, and feelings) from the sensations it registers externally. The fundamental differences between those signals appear to be: (i) self-generated signals are *predicted* in the brain (e.g., efference copy, corollary discharge^1–4^) and (ii) they are perceived to originate from a *location* in the body, i.e., self-location^5–7^. As we proposed previously^8^, these predictive processes may allow the brain to recognise the (bodily) self early on and to prioritise the processing of self-generated signals (i.e., endogenous attention system) over external stimulation (i.e., exogenous attention system). Therefore, when this mechanism fails, and these signals get muddled up, this may result in unusual perceptions (e.g., hallucinations, delusions^4,9,10^) and (out-of-body) experiences^11,12^.

Nested inside the body, the brain is naturally surrounded by three-dimensional space, in which events occur in an irregular yet irreversible succession from the past, present, into the future. To secure a successful interaction with its environment, the brain has evolved specific skills (e.g., building mental representations, internal models^1,2,4,13^, mirroring strategies^14,15^). Hence, these skills incorporate and largely reflect what it has come to learn or ‘statistically infer’ from its surroundings (see *Bayesian surprise*^16,17^). Yet, to be able to monitor, predict and control *what* it does, from *where,* and *when* relative to its surroundings, the brain has learned to reliably distinguish itself from it^2,4,17^ (see *inverse problem*, Ch. 1^13^). This ability, i.e., to perceive and monitor self- versus external actions, made it possible to fine-tune and guide more complex interactions (see *cognitive control*^18^). Unlike reflexive and automatic behaviours, we argue, this enabled human beings to evolve into highly specialised ‘agents’ that use purposeful actions to manipulate their environments and satisfy their needs (e.g., food, homeostasis, procreation^19^). Sensing control or ‘agency’ in voluntary actions appears so critical to human functioning that, when it fails, this not only hampers one’s perceptions and actions, but also one’s sense of self^20–23^. Undoubtedly, you are *you*, and your actions, feelings, and thoughts are *yours*, and not someone else's. Yet, when you lose the basic ability to recognise them, it becomes increasingly difficult to control and sensibly report on those things that make you *you* (i.e., what you do, feel, and think). This may have such profound ramifications that, at a certain point, you might be unable to separate what happens in reality from what is simulated in your mind (i.e., *psychosis*^20–23^). Thus, an inability to predict and monitor purposeful actions (also see *major depression*^24^*, movement disorders*^25^*, dementia*^26^) fundamentally impacts human functioning, causing confusion and distress in those individuals struggling to maintain a coherent sense of self.

Although it is still unclear how the brain constructs a coherent self, two senses are typically implicated: (i) is it *Me* or *my body* doing something, i.e., body-ownership^27,28^; and (ii) am *I* responsible or in control, i.e., sense of agency^27,29^. What has often been overlooked, however, is that humans commonly perceive that their actions, feelings, and thoughts originate from a *location* inside them self (see above)^5–8^. Perhaps coding such signals to a centre point of origin (typically to the body) evolved as an early adaptive strategy of the brain to recognise and keep track of itself relative to its surroundings. The first causal clues of such an embodied mechanism came from clinical reports^30–33^ describing out-of-body experiences (OBEs)^11,12^. Patients with focal epilepsy in the right temporoparietal junction, unusually, reported feeling located in their mind but not in their body^32^; whilst other studies confirmed that OBEs can be induced by electrically stimulating the right angular gyrus^11,30,33^ and when sensory signals are manipulated in full-body illusion (FBI) paradigms^34,35^. A typical FBI is passively induced by the experimenter, whilst the participant is required to remain motionless and still^12^. Through a Virtual Reality (VR) headset, people typically observe a virtual body located at some distance in front of them. For a period of time, the observer is passively stroked (e.g., with a brush on the back) whilst they see the virtual body being stroked at the same place and time. Because what is felt and seen is matched, this gives observers the false impression that what is experienced is happening in front of them. As proof of concept, they often report themselves to be closer to the virtual body than their actual location^34,35^ (this was coined ‘proprioceptive drift’^12^). But FBIs do not resemble everyday situations in which we can move voluntarily.

Altered cases of agency reveal that voluntary movements provide the brain vital clues to maintain a coherent self (e.g., *I* am doing that; *I* observe that my hand is moving^3,36^). They also establish a sense of place and time from one moment to the next (*I* am doing that *here* and *now*, *I* rush through the station to make my appointment; also see *intentional binding*^37^). Movement restrictions thus make it difficult for the brain to discriminate self- from external signals, preventing it from recognising (i.e., predicting) what signals belong to itself and which signals can be attributed (i.e., localised) to the body or not. This, we argued^8^, essentially tricks the brain into believing that the processed signals originated from somewhere other than the body. A failure to predict self- from external signals, an *agency dysfunction*, might thus underlie the dissociations seen in psychosis (e.g., hallucinations & delusions)^4,9,10^ and OBEs. A recent study provided the first clear causal evidence in support^8^. It was confirmed that FBIs can be enhanced by electrically stimulating the right angular gyrus. This not only hampered people’s ability to locate themselves, but also obstructed them from discerning self- from other perspectives on a simple computer task. A questionnaire further revealed that the reported dissociations were experienced as a loss in sense of agency. These new findings give insights into the relationship between mind and body and question if FBIs can be induced under conditions that allow people to move.

Four recent studies incorporated real-time movements in VR-simulations^38–41^. In two studies, people watched an avatar mimicking their body’s movements through a virtual mirror^38,39^. In two other studies, people made limited or partial movements aiming to self-induce an FBI. Through the headset, they either observed themselves stroking their own neck whilst sitting down^41^ or they physically moved virtual body-parts projected in front of them whilst standing^40^. All four studies found that synchronous movements elicited ownership of the virtual body (-part); but, irrespective of the range of movement, no shift in self-location towards the virtual body was found^40^. Perhaps the illusion may have been prevented this time because the brain could infer its location from movement. To test this, we will step-wise examine if healthy adults can perceive a human-sized avatar in a third-person game as their own.

## Results

**Figure 1:**
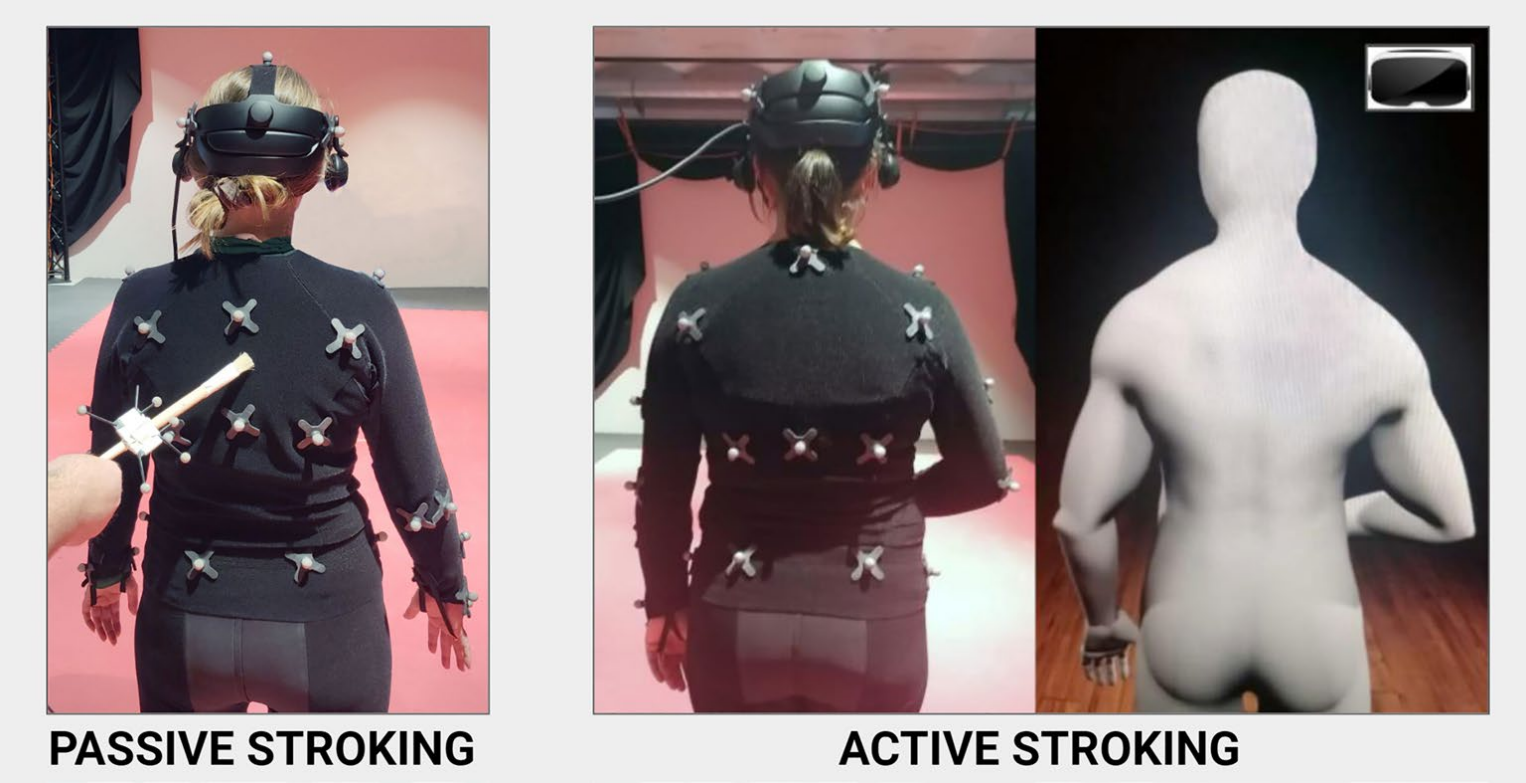
**Passive (Part A) versus active (Part B) full-body illusion.** Three-stage full-body Illusion (FBI): ‘Passive stroking’ (no motion control) versus ‘Active stroking’ (partial motion control). In Part A, a standard, passive FBI was recreated in virtual reality: participants were stroked on the back with a brush whilst simultaneously observing a virtual body being stroked on the back (5 min). Because what is ‘felt’ and ‘seen’ is matched this creates an out-of-body illusion^34,35^. In Part B, participants stroked themselves on the belly with their dominant hand whilst they observed the virtual body stroking itself on the belly with the same hand (5 min); can participants self-induce an illusion^41^ cf. ^40^ or does the illusion break down when the brain can predict its location from movement? See Supplementary materials: identical results were obtained when belly- vs. neck-stroking was compared in five individuals showing sufficient overlap in their avatar’s hand- and face models.

**Figure 2:**
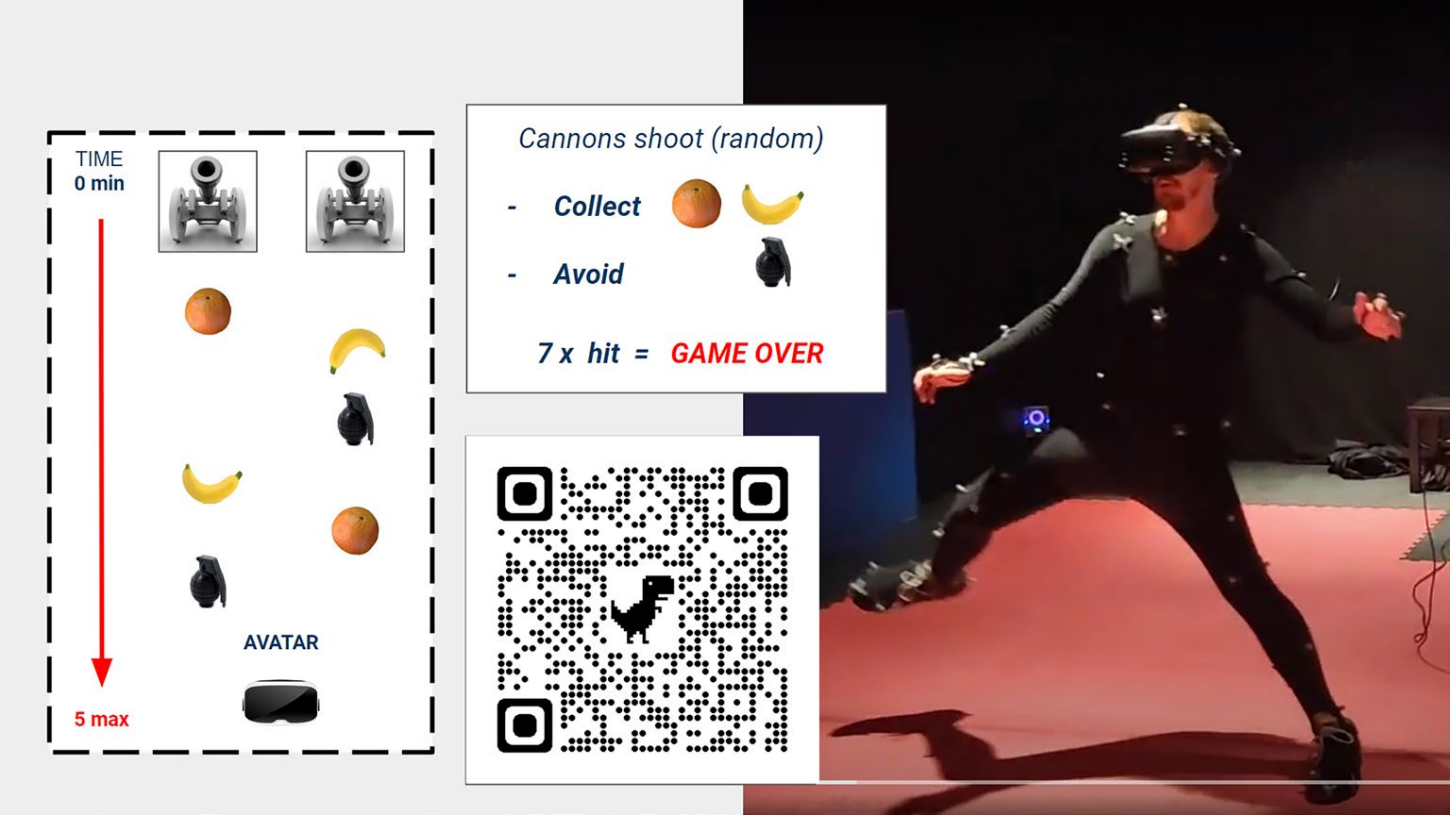
**Immersed (Part C) full-body illusion.** Three-stage full-body illusion ‘Part C Immersive Game.’ By physically moving their body around participants real-time controlled a human-sized avatar (i.e., ‘motion capture’ ensured that the virtual body moved as an exact copy of them). Their task was to collect fruit versus avoiding their avatar, located in front of them, from being hit by the occasional hand grenade. Two centrally positioned virtual cannons standing about ten metres away randomly shot fruit (4/5) and hand grenades (1/5) at them; a tone indicated success or failure. The player’s score and health were displayed in the playing field. The game ended after the avatar was hit seven times or automatically after 5 min. It was expected that the gameplay would motivate players to mentally align with their avatar’s position (see agency in *action observation*^52–54^). Scan QR code for a video-demonstration (note: the delay in the video projection is not in the actual footage, i.e., what gamers see through the VR-headset).

The experiment had three parts, see Figs. 1 and 2: first, (A) a standard (passive) full-body illusion (FBI) was recreated in VR; then, (B) we measured the effect it had when people made simple, voluntary movements, i.e., does it break the illusion?; lastly, (C) this setting was extended to an immersive game in which they real-time controlled a human-sized avatar in third person. In Part A-C (i.e., no motion control > partial motion control > full motion control) the avatar was observed from behind, resembling an OBE. Forty healthy adults (11 ♀, 29 ♂; Mean Age = 34.05, SD = 9.97) controlled for potential confounding factors (e.g., susceptibility, neuro- or psychological disorders, vestibular- and angular gyrus dysfunction) took part in the study (see Table S1). Half of them completed the passive (Part A) versus active (Part B) FBI before playing the game (ABC; 5 ♀, 15 ♂; Mean Age = 32.05, SD = 10.23); whilst the other half did the opposite (BAC; 6 ♀, 14 ♂; Mean Age = 36.05, SD = 9.55). Two participants were excluded: one had insufficient depth perception (♂ ABC-group; *n* = 20) and one session was omitted because of unforeseen technical issues (♂ BAC-group; *n* = 20). Occasional outliers and missing values (< 1% data points) were replaced by mean values (Part A: 1x  missing ‘Ordinal Time’; Part C: 1x ‘Estimated Time’, 1x ‘Play Score’ and 1x ‘Play Time’) or a cut-off score (‘Estimated Time’ 1x  Part A; 2x Part B). Statistical analyses were performed in SPSS v25.0 and reported under a 0.05 significance level.

### Exit-interviews

The *FBI Exit Interview* consisted of 15 items (incl. three control items), answered on 5-point Likert scales ranging from *‘1* = *Strongly Disagree’* to *‘5* = *Strongly Agree.’* The items measured altered self-location or *‘Displacement’*, *‘Self-Identification’* (i.e., body-ownership) and *‘Sense of Agency’* (see Supplementary materials). In the passive FBI (Part A), the displacement was perceived as: (i) a *“loss of control”* (Item 14 reversed; Agency; *n* = 28); followed by (ii) an identification with the virtual body (Items 5, 6, 10, & 15; Self-identification; *n* = 17); and (iii) a dissociation from the own body *“I felt a shift out of my body towards the virtual body”* (Item 2, Main Displacement; *n* = 13). A large subset also reported (iv) a regain in sense of agency and adopting a first-person perspective in the illusion: *“I felt as if I could stand up and walk away with the virtual body*” (Item 11; Agency; *n* = 15). In contrast, the active FBI (Part B) was characterised by: (i) a high sense of agency *“I felt in control of the virtual body”* (Item 14; *n* = 39); and (ii) no displacement (Items 2, 4, 7, 12 & 13; Displacement Total *M* = 76; *n* = 33). A large subset even reported (iii) no identification with the virtual body: *“I felt like someone else’s body was in front of me”* (Item 10; *n* = 11). The immersive game (Part C) was perceived as: (i) high in sense of agency (Item 14, *n* = 38; Item 8 reversed, *n* = 33; Item 11, *n* = 19); but also (ii) high in self-identification: *“I felt that the virtual body was my body that was moving”* (Item 5; *n* = 25). In the testing results, the total item scores are combined to a “*Total FBI Interview score”* (excl. control items 1, 3 and 9).

The *Game Exit Interview* consisted of 10 items, answered on an 11-point rating scale ranging from *‘0* = *not at all’* to *‘10* = *yes, I fully did’* (see Data S1). Contrary to the passive FBI (Part A), in the game (Part C) participants did not report a proprioceptive drift in self-location (Item 5; *M* = 3.48) nor a dissociation from their body (Item 3; *M* = 1.75). This was confirmed in their testimonies (i.e., two open questions asking to: (i) describe the displacement; (ii) comparing part A–C, see “Materials and methods”): in the passive illusion the displacement was described as a shift out of the body (Item 2) that felt uncontrolled (Item 14), and people identified with the virtual body (Item 5, 6, 10, and 15); whereas, in the game the displacement was described as a mental shift in self-location (i.e., gamers psychologically aligned with the avatar) that was self-directed (Items 8, 11 and 14). They reported: *“The game had little to no displacement but I strongly felt connected and in control of the avatar*;” and *“When I was focusing on catching fruit, I could ‘zoom in’ on the virtual body.”* Conversely, on the active FBI (Part B) they reported: *“it felt like watching a mirror image”*; and *“it made me focus on my own movements, which gave a connection with my body.”*

### Three-stage full-body illusion (FBI)

Statistics pooled over the experimental conditions revealed that the average self-reported displacement (Explicit measure) was 47.4 cm in Part A (passive FBI), -0.5 cm in Part B (active FBI), and 47 cm in Part C (immersive game). Participants reported that the displacement occurred *‘After a while’* (*n* = 16 Part A; *n* = 13 Part C) or 3 min (*M* = 2.6, *SD* = 0.9), which happened faster in the game (*M* = 1.5 min, *SD* = 1.9) than the passive FBI (*M* = 2.8 min, *SD* = 1). The displacement was experienced *‘Many short times’* (*n* = 8 Part A; *n* = 6 Part C), followed by *‘Continuously’* (*n* = 6 Part A; *n* = 4 Part C) and *‘Once shortly’* (*n* = 2 Part A; *n* = 3 Part C). Eighteen of the 40 participants reported no displacement (45%).

The effectiveness of the illusions (Part A-C; main results) were measures in four ways: both (i) implicit (blindfolded) and (ii) explicit (self-reported) ‘proprioceptive drift’ measures were taken (i.e., *‘Implicit Displacement’* and *‘Explicit Displacement’*); (iii) *‘Total FBI Interview’* scores were calculated (see above); and lastly, (iv) participants estimated how long each part took in minutes (i.e., *‘Temporal Binding,’* not to be mistaken for ‘intentional binding,’ measured salient sensory events and thus was estimated to correlate with the shifts in self-location). Further see *Procedure and tasks* & *Statistical Analysis*. A series of split-plot ANOVAs indicated no significant task-order effects *F* < 1. Therefore, the effects of the different levels of motion control (Part A-C) could be compared within one group (*n* = 40). A one-way (repeated measures) ANOVA performed over *‘Explicit Displacement’* found a large significant main effect of motion control on self-reported displacement, *F*(2.1,80.2) = 7.8, *P* = 0.001, ηp^2^ = 0.17. Mauchly’s test indicated a violation of sphericity over repeated tests (χ^2^(5) = 70.2, *P* < 0.001) and the *F*-test was corrected using Greenhouse–Geisser estimates (ε = 0.69). A second one-way ANOVA performed over ‘*Total FBI Interview*’ also confirmed a large significant main effect of motion control on experienced displacement, *F*(2,78) = 16.2, *P* < 0.001, ηp^2^ = 0.29; as well did a third one-way ANOVA performed over ‘*Temporal Binding*’, *F*(2,78) = 7.7, *P* = 0.001, ηp^2^ = 0.17. However, a fourth one-way ANOVA performed over *‘Implicit Displacement’* found no main effect of motion control on proprioceptive drift, *F*(2.7,103.6) = 0.6, *P* = 0.579, ηp^2^ = 0.02. Again, Mauchly’s test indicated a violation of sphericity over repeated tests (χ2(5) = 11.7, *P* < 0.05) and the *F*-test was corrected using Huynh–Feldt estimates (ε = 0.89). Likewise, a positive correlation was found between the dependent variables, *r*(39) > 0.4, *P* = 0.01; except for *‘Implicit Displacement’*, *r* = 0 one-tailed Bonferroni corrected.

**Figure 3:**
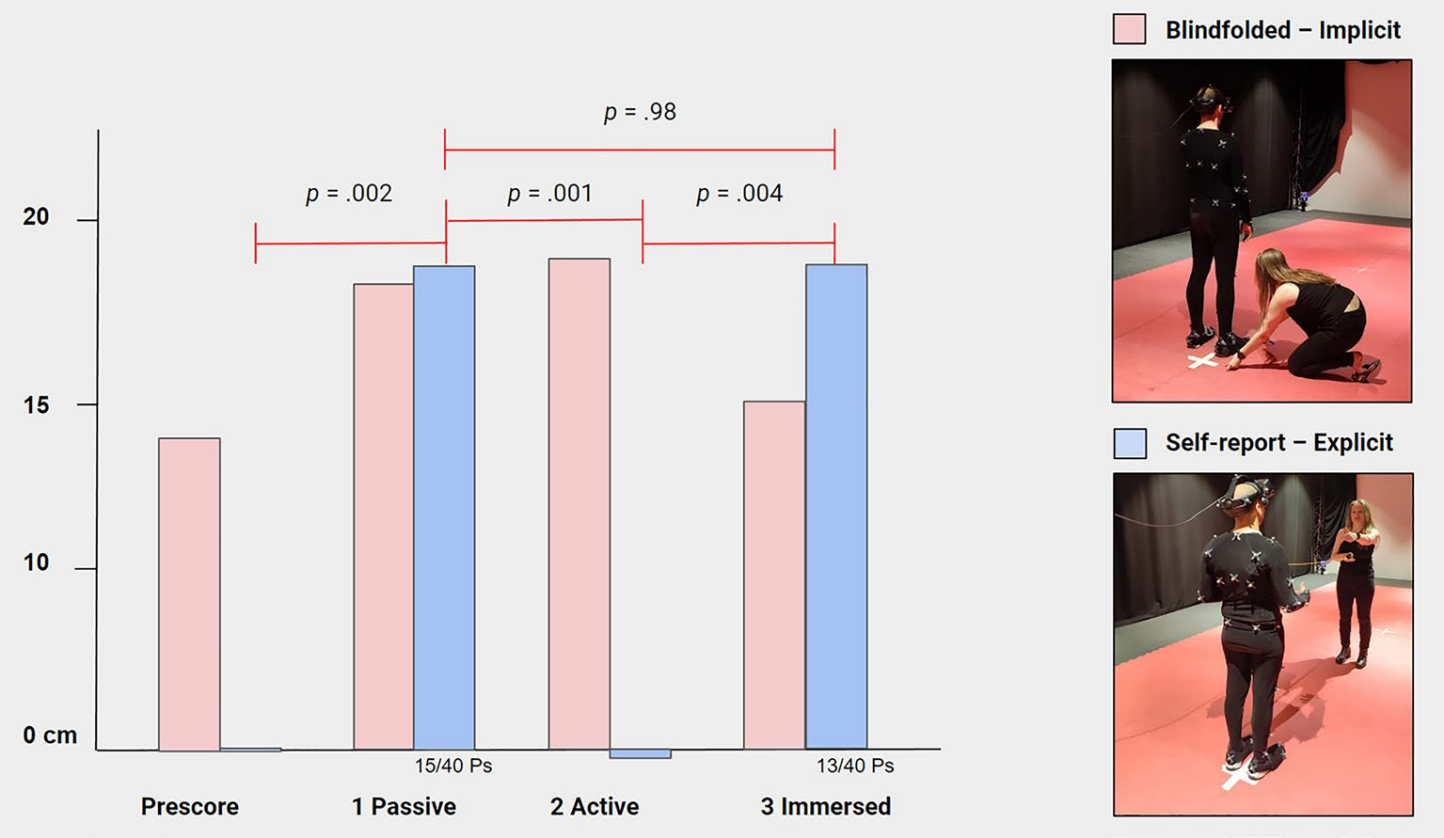
**Implicit (blindfolded) vs Explicit (self-reported) Proprioceptive Drift. **Displacement results in centimetres (cm) of ‘Implicit’ vs ‘Explicit’ methods to sample shifts in self-location, i.e., ‘Proprioceptive Drift.’ Three ascending levels of motion control are shown: (1) passive full-body illusion; (2) active full-body illusion; and (3) immersive game (*n* = 40). Implicit (standard) method: after the illusion participants are shortly blindfolded and passively displaced one metre back; they are asked to walk back to their original location; the difference in cm between the participant and their original location (white cross) is measured. Explicit method: after the illusion participants are asked to point out on a measuring tape if they felt a shift in self-location counting 0 cm from their body to 200 cm, the experimenter. Participants (Ps).

**Figure 4:**
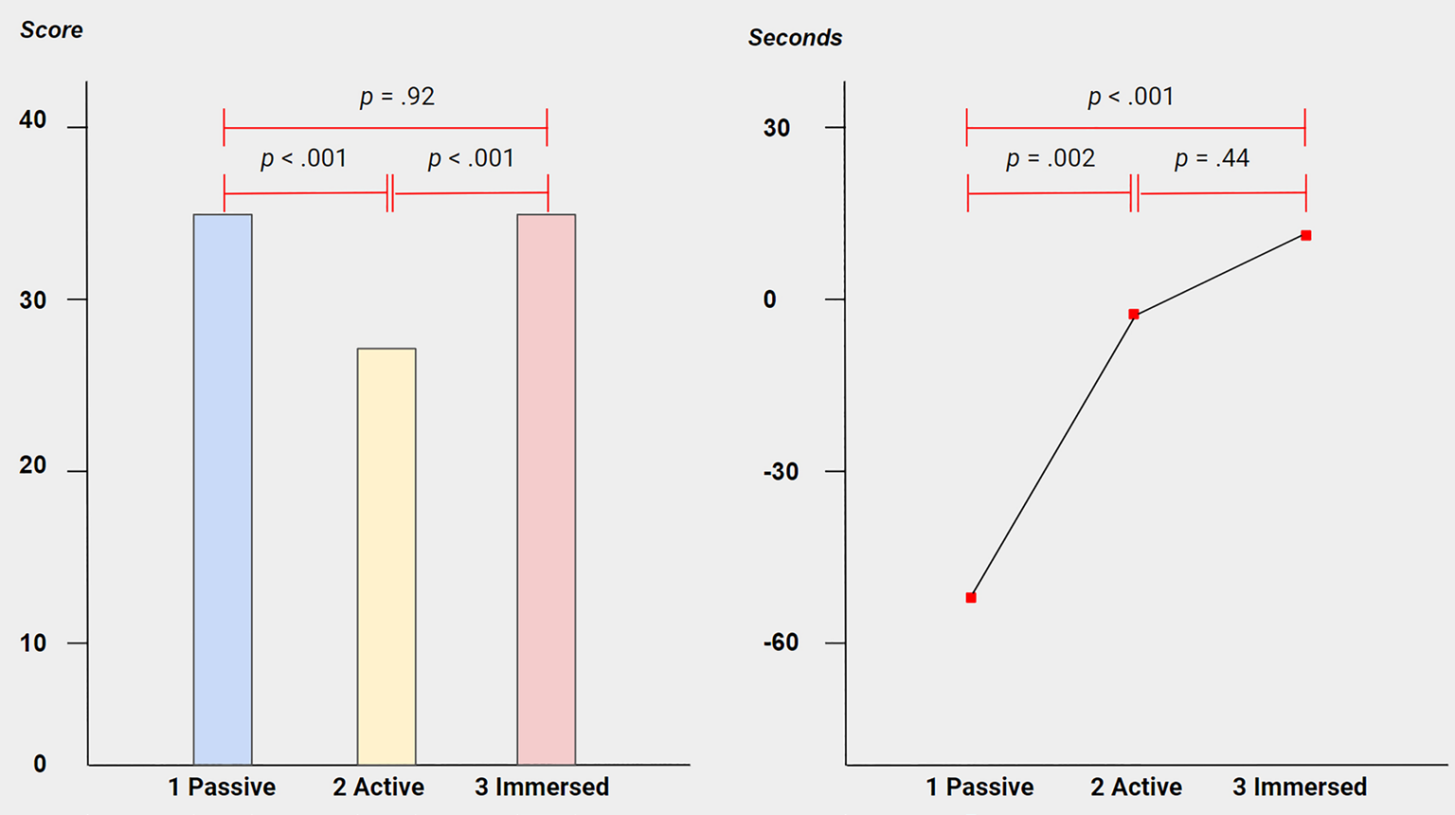
**FBI Total Exit Interview results and Temporal Binding results. **Mean ‘FBI Total Exit Interview’ scores and mean ‘Temporal Binding’ in seconds for the three ascending levels of Motion Control: (1) passive full-body illusion; (2) active full-body illusion; and (3) immersive game (*n* = 40). Temporal Binding: estimated time minus real time.

Four series of paired-samples *t*-tests (i.e., one for each dependent variable) were performed to check for the direction of the effects, see Table 1, Figs. 3 and 4. As expected, a first series of *t*-tests performed over *‘Explicit Displacement’* found more self-reported displacement in the passive FBI (*M* = 17.8, *SD* = 33.7) than at baseline (i.e., pre-test score 0), *t*(39) = − 3.3, *P* = 0.002; and during the active FBI (*M* = − 0.5, *SD* = 9.3), *t*(39) = 3.6, *P* = 0.001. However, no significant difference was observed between the passive FBI and the game (*M* = 17.6, *SD* = 36.9), *t*(39) = 0.02, *P* = 0.981. Similarly, there was more self-reported displacement in the game than at baseline, *t*(39) = − 3, *P* = 0.004; and the active FBI, *t*(39) = − 3, *P* = 0.004. A second series of *t*-tests performed over ‘*Total FBI Interview*’ gave identical results: as expected, more displacement was experienced in the passive FBI (*M* = 34.3, *SD* = 9.4) versus the active FBI (*M* = 27.9, *SD* = 6.6), *t*(39) = 4.8, *P* < 0.001. However, no significant difference was observed between the passive FBI and the game (*M* = 34.4, *SD* = 9), *t*(39) = 0.1, *P* = 0.932; also more displacement was experienced in the game versus the active FBI, *t*(39) = − 4.6, *P* < 0.001. A third series of *t*-tests performed over ‘*Temporal Binding*’ confirmed that the passive FBI was estimated about 1 min shorter (*M* = − 52.5, *SD* = 88.3) than the active FBI (*M* = − 5.3, *SD* = 99.4), *t*(39) = − 3.2, *P* = 0.002; and the game (*M* = 9.7, *SD* = 73.2), *t*(39) *p* = − 4, *P* < 0.001. This time, no difference between the active FBI and the game was observed, *t*(39) = − 0.8, *P* = 0.44. Finally, a last series of *t*-tests performed over *‘Implicit Displacement’* did not find any significant differences in proprioceptive drift between the conditions: Passive FBI (*M* = 17.2, *SD* = 18) versus baseline (*M* = 14, *SD* = 19.4), *t*(39) = − 1, *P* = 0.321; versus active FBI (*M* = 18, *SD* = 17.4), *t*(39) = − 0.3, *P* = 0.789; versus game (*M* = 15.4, *SD* = 20.7), *t*(39) = 0.7, *P* = 0.48. Active FBI versus baseline, *t*(39) = − 1.2, *P* = 0.234; versus game, *t*(39) = 0.9, *P* = 0.399. Game versus baseline, *t*(39) = − 0.3, *P* = 0.742. Moreover, a mean displacement of 14 cm was found at baseline (pre-test).

**Table 1:** Displacement results (*M*; *SD*) in three levels of Motion Control.

Displacement measure	Motion control							
	Pretest		1: passive		2: active		3: immersed	
	*M*	*SD*	*M*	*SD*	*M*	*SD*	*M*	*SD*
Explicit (cm)	0	0	17.8	33.7	− 0.5	9.3	17.6	36.9
Implicit (cm)	14	19.4	17.2	18	18	17.4	15.4	20.7
Exit-interview	–	–	34.3	9.4	27.9	6.6	34.4	9
Binding (sec)	–	–	− 52.5	88.3	− 5.3	99.4	9.7	73.2

Displacement results in three ascending levels of ‘Motion Control’: 1. passive full-body illusion; 2. active full-body illusion; 3. immersive game (*n* = 40). Format ‘Displacement measure’: Explicit (self-report in centimetres); Implicit (blind-folded in centimetres); Exit-interview (‘Total FBI Interview’ score); Binding (‘Temporal Binding’: estimated time minus real time in seconds). Mean (M); Standard Deviation (SD). In the active full-body illusion (Explicit) only two participants reported a small shift towards the virtual body, whilst three other participants reported a shift away from the virtual body (*M* = − 0.5).

## Discussion

This study examined if motor predictions in the brain enable healthy human adults to infer self-location and to distinguish their body from the environment. By uniquely combining a VR-setup with full-body motion capture, a full-body illusion paradigm (FBI) was developed with three motion control conditions: (A) a standard, passive FBI in which people had no motion control; (B) an active FBI in which they made simple, voluntary movements^40,41^; and (C) an immersive game in which they real-time controlled a human-sized avatar in third person. In all conditions (A-C) the avatar was seen from behind, resembling an OBE. Comparisons between implicit and explicit measures revealed a causal relationship between (i) motion control (prospective agency), (ii) self-other identification, and (iii) the ability to locate oneself. A loss in sense of agency was reported when movement was restricted and people experienced a shift in both self-location and self-identification towards the virtual body. This did not happen when they were (to some extent) able to voluntarily move: people can recognise their movements in a game avatar and psychologically align with it (action observation); but they do not normally lose a sense of place (self-location), time (temporal binding), nor who they are (self vs. other), because self-action appears to code the self to a physical location in space and time. Thus, the present results confirm that motor predictions are salient cues for the brain that not only provide a sense of control in purposeful actions, but also recognition of the self in space and time (see bidirectional neural mechanism below)^8^.

Similar to previous studies^38–41^ we expected that people would report (i) ownership of the external body; but would also (ii) sense agency of its movements (i.e., you observe a match in movements and conclude that *you* must be making those movements)^42^. Foremost, this was expected to happen in the game-setting where there was a clear motivation to psychologically align with the avatar. This is precisely what was found, but some measures failed to distinguish between a proprioceptive drift in self-location (see passive illusion) and a self-directed mental shift in self-location (see game). Participants reported this distinction in their testimonies, which was also found in the temporal binding results and in the game exit-interview: an involuntary shift in self-location was apparently so eventful that the passive illusion and not the game (i.e., in which they had full motion control; cf. intentional binding^37^) was experienced as significantly shorter in duration; similarly, the game exit-interview confirmed that (iii) people’s ability to locate themselves remained unaffected when they voluntarily moved; nor did they feel dissociated from their physical body whilst gaming. This is in line with previous findings: when participants made voluntary movements attempting to self-induce an illusion, Kondo et al*.*^40^ found neither direct nor indirect evidence of a shift in self-location (i.e., in proprioceptive drift, self-reports, and skin conductance responses).

It needs to be critically noted that Swinkels et al*.*^41^ reportedly succeeded in self-inducing an illusion. However, they failed to present results in direct support (i.e., they reported on the suppression of touch and on delayed responses) and their findings appear to be biased in some other way. As Lush et al*.*^43^ argued, to a great degree *individual susceptibility* (or suggestibility) determines the effectiveness of an illusion. This is a known issue of body transfer illusions that could have negatively impacted our results had we not controlled for this (contrary to some previous studies we predicted that voluntary movements would block the illusion from manifesting). Swinkels et al*.*^41^ did the opposite: susceptible individuals in whom the stroking induced an illusion were selected for the study. Yet, certain aspects of susceptibility are linked to high schizotypy personality traits^44^ and, more precisely, to a diminished sense of agency and self-recognition (see “Introduction”). Thus, making a limited range of movements whilst otherwise remaining motionless (i.e., sitting down, stroking one’s neck confined to a small area) may not have elicited sufficient efference copy (i.e., sensory attenuation) in those individuals to block the manifestation of the illusion. Kondo et al*.*^40^ tested a larger range of limb movements and did not find such evidence (see above). Hence, to obtain more conclusive results, this study compared: (i) different levels of motion control; whilst (ii) controlling for individual susceptibility (see “Materials and methods” and/or *the Phenomenological Control Scale*^43^). Once these confounding factors were ruled out, voluntary movement again blocked the illusion from manifesting, as in traditional FBI-paradigms^12^.

A current limitation of the methods is that the implicit measure (blindfolded) gave no significant results in contrast to the other measures that all showed large effect sizes (ηp^2^ > 0.14). Yet, it remains unclear to what degree a positive ‘difference score’ represents a true shift in self-location or that the illusion succeeded. Such a score may, in part, reflect a misjudgement from a convoluted walking back-procedure (see Method); or represent individual differences in the ability to perform the task^45^; or a difficulty in judging distances in VR (a known simulation problem). Thus, a shift in self-location cannot automatically be deduced from this measure. A recent review also indicated reliability issues using this method: a large spread in scores between studies and possibly a shift at baseline (see Fig. 4 in^45^). Certainly, other implicit measures like biological markers (e.g., heart rate elevations; skin conductance responses) give a good indication. But because phenomenological experiences are inherently subjective, this makes it difficult to sample to what extent an illusion had succeeded unless this is also explicitly confirmed (e.g., with standardised self-reports). For these reasons, the present study compared various implicit *and* explicit measures in a carefully controlled repeated design (incl. pre-test baseline scores and not congruent vs. incongruent conditions that would complicate such a design). This novel approach gave some critical insights (see above) and confirmed that the implicit method was sensitive to false positives (i.e., measuring a shift at baseline and high intra- and inter-subject variability).

An important contribution of this study was demonstrating that a full-body illusion can unlikely be provoked under natural conditions (i.e., letting healthy individuals observe themselves make voluntary movements). For a fact, people do not ‘displace themselves’ when they gaze into the mirror whether they make movements or not. Humans can recognise themselves in a shop window and can often instantly tell that it is them from their movements. But they do not normally lose a sense of where (self-location) or who they are (self/other) when doing so. The neural processes involved are much too intricate and refined: efferent signals produced in voluntary movement are functionally integrated into motor schemas in the brain. They involve anatomically overlapping structures in the (pre)motor and parietal cortices that, once activated, suppress or ‘cancel out’ feedback stemming from the sensory systems (i.e., reafference). This ensures a rich, unified experience when the body is in motion with a current dominance over other signals^3,36^. Clever experiments, like the ones of Tsakiris et al*.*^3^, confirmed that efferent signals provide vital clues of self-action to the brain. They showed that healthy volunteers were better at judging whether a hand seen on a TV-monitor was theirs when they indirectly yet voluntarily (with a lever press) caused their hand’s index finger to move than when they either viewed their finger being passively moved or someone else’s finger moving. Because of this setup, the only means to discriminate between the conditions were (the predictions of) the self-generated movements. Hence, by prospectively monitoring them, participants could infer their agency and distinguish themselves from other stimuli. In other words, the body and the brain are deeply intertwined in voluntary action, making it unlikely that healthy human adults can misidentify another body as their own (see game). Nonetheless, this study confirmed that they can: (i) psychologically align with another person and perceive agency in observing actions (see mirror neuron system^14,15^); and (ii) suppress crude bodily sensations when prioritising tasks that require direct attention^41^. But healthy adults can neither physically nor psychologically disconnect from their body and its location when engaged in voluntary action.

Predictions of self-actions are crucial to maintain a coherent self. In our thoughts and dreams we may (at will) disconnect from our bodies when not engaged in action. Yet, altered cases of agency (e.g., OBEs, psychosis) caution us that these processes can sometimes easily become disrupted. This first came to light in clinical reports revealing that specific cortical structures could be excited to temporarily evoke such perceptions in the healthy brain (i.e., by electrical stimulation) and the abnormal brain (i.e., in epilepsy). More recent studies^20–23^ demonstrated that psychosis symptoms (e.g., hallucinations, delusions) are likely caused by an inability to predict and monitor self-generated signals (i.e., prospective agency). It was found that, in the preparation of a voluntary action, the angular gyrus (i.e., exogenous attention), whose activity is normally suppressed by the frontal control network (i.e., endogenous attention, see proposed bidirectional mechanism^8^), atypically continued its function^23^. This, we argued, may have caused an alteration in processing self- versus external signals, and an opportunity for unusual perceptions to arise. Efference copy, i.e., predictions of voluntary action, gives the brain instant clues as to the origin of self-generated signals (i.e., self-location). This may be an efficient way for the brain to early discriminate the body from its surroundings. Likewise, convergent evidence suggests that *doing* and *thinking*/*observing* that same thing are processed similarly in the brain (see mirror neuron system^14,15^). Yet, what appears to distinguish the two situations is *the origin* of the voluntary action (i.e., self-location). This may be a necessary step for the brain to differentiate the body from its environment.

In closure, a failure to discern (the origin of) self versus other signals and by extension what is real from what is imagined (i.e., psychosis) is seen in psychopathology (e.g., schizophrenia, intoxication, post-traumatic stress) but also in neurodegeneration (e.g., dementia, delirium, movement disorders; see “Introduction”). Because the underlying neural mechanisms appear fundamentally tied to the brain’s motor control function, the importance of keeping these networks strong, especially in old age, becomes exceedingly clear: when individuals lose their sense of agency, they lose their sense of self and who they are in the long-term. This cannot be avoided by stimulating one’s mental capacities alone. This is where embodied games may offer great future benefits. In our game, people real-time controlled a human-sized avatar in third person; a hugely immersive and engaging experience (9/10 for ‘fun to play’). Unlike first-person simulations, this did not evoke debilitating adverse effects, i.e., simulation sickness^46,47^. Hence, in the future embodied games could more systematically be used to target and strengthen the brain’s control networks in a fun and relaxed setting. This could offer non-invasive treatment options for a growing group of vulnerable individuals in the clinic or (nursing) home. Motion capture can also be integrated into rehabilitation techniques (e.g., to treat nervous system injury, phantom limb pain). A technical advantage of doing this is that any type of motion (incl. complex limb movements) can be simulated with millimetre precision. Lastly, in a computer generated environment basically anything can be customised to fine-tune, personalise, and augment the therapeutic setting (e.g., visual cues can offer feedback and guide individual therapy). Mirror therapy^48^ has shown robust effects in treating phantom limb pain, yet, considering the above techniques, future possibilities seem endless.

## Conclusion

Self-action (i.e., motor predictions in the brain) enable healthy human adults to infer self-location and to distinguish their body from (other agents in) the environment. Inferring self-location may have evolved out of a need for the brain to recognise and monitor self- versus other purposeful actions and their outcomes (i.e., prospective agency).

## Materials and methods

### Participants

Forty-two healthy naive adults with normal vision, hearing and vestibular function volunteered for the experiment (31 ♂, 11 ♀). Each received a gift voucher for their participation. Participants were recruited through study advertisements posted on the online community pages of the local work offices (Ghent, Belgium). Sample size was set based on *n-*sizes reported in similar studies^40^ (for power calculations^41^). It consisted of two significantly large groups (2 × *n* = 20) to rule out transfer- and task order effects (mixed design). Prospective participants were pre-screened online for (i) VR-safety^47,49^, (ii) any neuro- or psychological disorders, (iii) visual and vestibular problems (related to processing 3D content), and (iv) susceptibility to body transfer illusions^44^. Table S1 lists all exclusion criteria. Susceptibility was assessed with the 20-item *time Perceptual Aberration Scale* (tPAS) and identified no individuals scoring within the top 10% of the scale. Past research found a strong correlation between the tPAS and the Perceptual Aberration Scale (i.e., measuring vulnerability to schizophrenia spectrum disorders), and between high tPAS scores and right angular gyrus dysfunction^44^. Four participants reported prior out-of-body experience unrelated to medical injury or near-death experience. Participants gave written informed consent and approved the use of identifiable images. The study was approved by Le Comité d'Ethique of the Université libre de Bruxelles (116/2020) and the Human Research Ethics Committee of the Queensland University of Technology (1800000632) and conducted according to the Declaration of Helsinki (WMA, version October 2013).

### Three-stage full-body illusion paradigm

A full-body illusion paradigm (FBI) was designed to systematically analyse what role sense of agency has on people’s ability to locate themselves, and if (predictions of) voluntary movements allow the brain to infer self-location and to distinguish the body from the environment. To create precise, high-quality motion simulations, real-time full-body motion capture (Vicon, Oxford, UK)^50^ was incorporated into a VR-setup (Valve Index)^51^. This way, VR-simulations with various degrees of motion control were created: (Part A) no motion control; (Part B) partial motion control; and (Part C) full-body motion control (Figs. 1 and 2). It was expected that: (i) people would report ownership of an external body that moves like them^38–41^; and (ii) would, retrospectively, report sensing agency over the avatar’s movements in Part B and C (i.e., you observe a match in movements and conclude that *you* must be making those movements)^42^. Foremost, this was expected to happen in the game-setting where there is a clear motivation to psychologically align with the avatar’s movements (see agency in *action observation*^52–54^ and *mirror neuron system*^14,15^). However, (iii) people’s ability to locate themselves should remain unaffected when the brain can properly infer the body’s location from movement^40^ cf.^41^.

### VR combined with motion capture

#### Valve index VR-setup

To create precise, high-quality motion simulations, we combined a VR-setup (i.e., the Valve Index excluding controllers)^51^ with real-time motion capture (Vicon)^50^, see Fig. S1. The Valve Index has a dual full-RGB LCD display with a respectable resolution of 1440 × 1600 per eye and a refresh rate of up to 144 Hz (0.330 ms ultra-low persistence global backlight illumination). The padded headset was fully adjusted to head size, face angle, and ear positioning; and the interpupillary distance (IPD) and eye relief (distance from lens to eye) were adjusted to accommodate for a persons’ field of view. The headset connected to the PC with a 5-m tether and 1 m breakaway trident connector cable (12 V power) that plugged into a 3.0 USB to DisplayPort 1.2 converter. Two SteamVR base stations 2.0 (160º x 115º field of view) were mounted high on opposite ends of the experimental area (4 × 5 m of 11 × 7 m total lab space) to set up the playfield for 360º positional tracking. The base stations (i.e., two infrared (IR) laser beacons) used Lighthouse technology to integrate 3D positional information (e.g., object's orientation, velocity, angular velocity). In simple terms, this meant that at about 50 times per second the base stations synchronously swept the room with alternating horizontal and vertical pulses and laser lines, reaching out to about 7 m in distance (23 feet). The SteamVR Tracking system used the timing between pulses and sweeps and simple trigonometry to track the location of each head- and controller sensor at ~ 1.5 mm precision and ~ 1.9 mm accuracy. This was accomplished at an update rate of 1000 Hz with very low latencies (2 ms). For system requirements see Supplementary materials.

#### Unreal engine simulations

This was the first time that full-body motion was captured and translated real-time to an avatar in a third person VR-simulation (note: a first-person simulation does not require full-body motion capture). This was previously impossible due to insufficiently advanced technology. The Valve Index, using SteamVR technology, requires a SteamVR plugin to be installed on the PC. Steam is an online video game digital distribution service (Valve, 2003)^51^ where you can buy, play, create, and discuss PC games within a vast global community (> 150 million users). Unreal Engine 4.26.2, a 3D computer graphics game engine^55^, was used to create and real-time render two simulations in VR: (i) one basic ‘Empty Room’ simulation (FBI Part A & B); and (ii) a similarly shaped ‘Game Arena’ for the Immersive Game (Part C), see Figs. S2 and S3. Although rendering large data volumes still poses a significant challenge, Unreal^55^ is currently the most powerful real-time 3D creation tool (free for non-commercial use). Because we used captured data (Vicon PC 1, see below) to real-time stream our participants’ movements to VR (VR PC 2, see above) this took up large amounts of memory and processing power. Therefore, any data that was live-handled by Unreal (e.g., environments, virtual characters, objects) had to be downsized to subjectively ‘good enough’ quality to avoid any unwanted jitters and latency problems and/or prevent the engine from crashing during simulation (e.g., implementing an Unreal MetaHuman as virtual character proved unfeasible). Hence, the most detailed simulated features of the game were: (i) the (movements of the) virtual character; and (ii) some movement- and rotation animations of the objects.

A standard Vicon Human Skeletal Mesh was used as a virtual character that was exported to Unreal and colour calibrated (i.e., removing any tracker lines by painting the mesh evenly grey and giving it a surface effect to reflect light; Fig. 1). It stood in the middle of the virtual room in front of the participant, and was illuminated from the back by two spotlights from each side to give it more depth information^56^. To create a set of photorealistic 3D-objects for the illusion (i.e., a paintbrush) and the game (i.e., a banana, orange, hand grenade and cannon) the free 3D modelling software Blender^57^ was used. The objects were later downsized and exported to Unreal. The ‘Game Arena’, e.g., consisted of simple line blocks stacked upon each other that, looking from the player’s point of view, gave the immediate impression of depth and space. Centrally located towards the back of the arena, about eight metres behind the virtual character, two 3D-modelled cannons (one a duplicate of the other) randomly shot fruit (4/5) or a hand grenade (1/5) at the virtual character. In Unreal Blueprint^55^ a construction script and event graph were built that ensured that objects were spawned and shot with different percentages. Once collected or hit, a specific sound (of similar duration) would play into the player’s ears communicating success or failure: two upbeat sounds for the fruit (success: collected!); and an explosion sound for the hand grenade (failure: hit!). Objects were automatically terminated after bumping into the character or the floor plane. Similarly, the player's ‘game score’ (i.e., a text box with three digits; seen top right visual field) and ‘health’ (i.e., seven 3D hearts; seen top left visual field) were scripted in Unreal to indicate positive and negative events: when a fruit was collected a digit was added to the score; whilst a heart was eliminated each time a grenade hit. Lastly, to ensure that the game remained fun to play it had three built-in difficulties: the spawning interval between objects (ISI) would decrease by 0.25 s when reaching 70 points (level 1 ISI = 1 s), 120 points (level 2 ISI = 0.75 s); and 180 points (level 3 ISI = 0.5 s). A hidden timer would stop the game when hit seven times with a hand grenade or after 5 min of playing.

#### Vicon motion capture

To develop a full-body illusion paradigm with different movement conditions, a virtual character was created in Unreal that (i) had about the same characteristics (e.g., body size, height) and (ii) mimicked participants’ movements with millimetre precision. To accomplish this, we used a tracking technique called ‘motion capture’ that mapped real-world limb movement onto a 3D model of a humanoid (note: the VR-headset and hand controllers have insufficient data points for this purpose). A Vicon Motion Capture System with a setup of 20 infrared Vero 2.2 cameras and one HD 1080p reference video camera (lab space: 7 × 11 m) collected detailed and reliable movement information from each participant (Fig. S1)^50^. The captured data was then used by Unreal to real time model movements onto a 3D humanoid standing virtually in front of the participant (Fig. S2). To let Vicon (PC 1) and Unreal (PC 2, see VR-setup) work together Live Link Plugin 1.5 for Unreal Engine 4 was downloaded that allowed Vicon to real-time stream the captured data to the Skeletal Mesh of a basic character in Unreal.

Vicon systems (used in the professional industries for over 35 years)^50^ rely on ‘passive optical motion capture.’ This is a technique that uses retroreflective markers that are strategically placed onto bodies or objects to best recover their original shape and/or body pose. The markers reflect the light emitted by small infrared strobes positioned around the camera lenses. To guarantee smooth and accurate full-body tracking, we used a standard Vicon character model of 57 data points for marker placement: 44 on the body (mocap suit); five on the VR-headset; and four on each foot (Fig. S1). In addition, a large paintbrush size 12 (3048 mm long × 15 mm wide) was equipped with five markers for use in the passive full-body illusion. Each Vicon Vero camera v2.2 is a fast, high-resolution (2.2 megapixel; wide: 98.1º × 50.1º tele: 44.1º × 23.6º field of view) device that uses infrared technology to track differences in light bouncing off from objects at 330 frames per second. Once reflected, Vicon’s optical tracking system uses this light to calculate the position of the markers with up to 0.017 mm dynamic accuracy within a three-dimensional space. The data are then live recorded at high speed (1000 Hz; 330 frames per second) and translated to the computer at an extremely low latency (1.5 ms). Vicon Shogun Live 1.4 was used to calibrate the system and participant prior to data collection. For system requirements see Supplementary materials.

### Procedure and tasks

The Vicon camera setup was calibrated daily prior to starting the experiment. In the middle of the 11 × 7 m laboratory space a white cross was placed on the floor indicating participants where to stand. The connection cord of the VR-headset was attached to a retractable extension cord reel hanging ~ 2.5 m above the cross, so that participants could move around freely whilst wearing the headset. After signing consent, participants were instructed to wear a motion capture suit and a VR-headset with five markers. The suit was then manually equipped with another 52 markers (total = 57), see Fig. S1. To guarantee smooth and accurate motion capture, a set of poses were performed (e.g., A-pose, T-pose) that allowed the standard humanoid in Vicon to be adjusted to the dimensions and shape of the participant. In Unreal, the height of the participant was inserted to match the camera’s viewing point to that of the virtual character. Short breaks were inserted between experimental parts A–C to allow participants time to rest.

#### Passive versus active illusion (Part A and B)

The first two simulations took part in the ‘Empty Room’ (Fig. S3) and involved (Part A) a passively induced FBI performed by the experimenter (i.e., the participant had no movement control) versus (Part B) a ‘self-induced’ FBI performed by the participant (i.e., the participant had partial movement control). In line with our hypothesis, the active FBI (Part B) was expected to function as a baseline condition to the passive FBI (Part A) (i.e., blocking the manifestation of the illusion). Hence, to control for transfer and task-order effects that might otherwise explain the results, half of the participants underwent the passive illusion first (AB-order) whereas the other half performed the active illusion first (BA-order). At the start of the experiment two pre-test baseline measures were taken. Participants stood behind the white cross when the ‘Empty Room’ simulation started. Some manual simulation checks were performed (e.g., adjusting avatar to participant height; streaming checks) and having observed the virtual body for some time, they were asked to close their eyes (i.e., to measure ‘*proprioceptive drift*’, or the absence thereof, before the illusion). The experimenter gently held the participant at the shoulders and asked to carefully walk back together, in the process, passively displacing the participant one metre behind. Subsequently, the participant was asked to walk back to their original location. The difference between the participant’s new location and their original location (i.e., the white cross) was measured in centimetres on the ground; ‘Implicit Displacement.’ For the second pre-test measure, they were asked to carefully lift up the VR-headset. They saw the experimenter standing in front of them holding up a measuring tape counting out 0 cm from their body (mid-thorax) up to 200 cm away from them (i.e., this was done to measure their ‘self-location’ before the illusion). They were asked to indicate their self-location on the measuring tape (e.g., ‘0 cm’ indicated inside their body); ‘Explicit Displacement.’ Afterwards, they put their headset back on.

Before the illusion started, participants were instructed to stand completely still throughout the procedure and focus on what happened in front of them without moving their gaze. A timer was set to 5 min (note: without the participant’s knowledge) whereupon the experimenter gently started stroking them with a brush at about 50 strokes per minute. Synchronously, the participant could observe the virtual body being stroked with a virtual brush at the same location. The stroking was situated on the upper part of the middle back over a total length of 15–20 cm^56^. Therefore, during a continuous period of 5 min participants ‘*observed*’ in front of them what they ‘*felt*’ happening to them. Immediately after the illusion, participants were again asked to close their eyes, and, following the same procedure, the experimenter would displace them one metre back and the measuring procedure would repeat: (i) the difference in location between the participant and white cross was measured (implicit displacement); and, this time, (ii) participants were explicitly asked if they at any point felt displaced towards the virtual body (or the virtual body towards them) and, if so, to point that out in centimetres on the measuring tape. Pointing ‘0 cm’ indicated no displacement; whilst everything else indicated a certain amount of displacement. They were instructed: *“there are no right or wrong answers; some people experience something while others don’t. An honest answer is simply required.”* Lastly, participants (iii) estimated how long the illusion took in minutes (see *‘temporal binding’* below). After this was noted down, Part B of the experiment could start: participants were instructed to gently stroke their belly with their dominant hand, whilst they synchronously observed the virtual body doing the same. The stroking situated above the navel-point over a total length of 15–20 cm from left to right. This part again lasted for 5 min and measurements i-iii were taken. Part C followed the same procedure.

#### Immersive game (Part C)

In this part participants performed a game, in which, for the first time, they could play their own avatar in third person. In other words, by making movements themselves, participants had one-on-one control over a virtual body that was proportionately the same as them. Before starting the ‘Game Arena’ simulation, participants were carefully informed of the task: *“collect as much fruit as you can with your avatar but avoid being hit by a hand grenade. After being hit seven times the game will stop.”* They were further instructed that they could use their entire body to collect objects (all ranges of movements were possible, e.g., sticking out hands and feet, jumping from the floor, ducking to avoid grenades). The objects simply had to collide with their avatar to count as a hit. However, because the VR-headset was not wireless and may become disconnected when players wondered off too far, in the game the white cross on the floor was substituted for a large horizontal line they were instructed not to cross. Lastly, because a third-person VR-game did not exist, it was anticipated to be somewhat challenging at first to use your entire body to collect/avoid objects and control an avatar positioned away from you. Therefore, first a test run was completed: participants took centre stage behind the line/white cross and the game started counting down from 10 s. Typically, it took 90–120 s before their avatar was hit seven times with a hand grenade and the game stopped. They were debriefed to check if they had understood the task / acquired a feeling of how to catch/avoid objects with their game avatar. This appeared to be quite intuitive for most, but if occasionally this turned out to be hard, it was explained to them that the objects would reach their avatar earlier in time because they were standing behind it, and thus at a *further distance*. This required them to make earlier-timed responses to catch/avoid objects and, so-to-speak ‘mentally align’ with the avatar rather than their own position. If that was clear, the *game session* could start, lasting 5 min if completed successfully. Measurements i-iii were taken and the participant was checked for symptoms of simulation sickness (see Table S2; using an adapted version of the Simulator Sickness Questionnaire^46,47^). Finally, they were placed behind a desk and completed two exit-interviews: an *FBI Exit Interview* (8 min); and a *Game Exit Interview* (2 min), see below. One experimental session lasted ~ 60 min.

In summary, the effectiveness of the illusion in Part A-C was measured in four ways: ‘displacement scores’ were taken before (pre-test score) and after (post-test score) the illusions. The standard way of doing this is to (i) implicitly scope participants’ perceived shifts in self-location or *proprioceptive drift*^45^, see above. This was also measured by (ii) *explicitly asking* if participants felt shifted towards the virtual body and to point that out on a measuring tape, as above. In addition (iii) participants estimated how long each part took in minutes (Part A-C). This measured *temporal binding* and was expected to correlate with the shifts in self-location: salient sensory information is grouped together in the brain, subjectively shortening perceived time (note: this measure should not be mistaken for ‘intentional binding’ that concerns the subjective shortening of time when actions are voluntary^37^; see Data S2)^58^. After the experiment (iv) *an exit-interview* was completed questioning participants about their experiences in each part (8 min duration). They indicated their agreement to three sets of 15 statements intended to measure displacement, e.g., *‘I felt I was in front of my body,’* scored on 5-point Likert scales ranging from *‘1* = *strongly disagree’* to *‘5* = *strongly agree,’* see Table S3 and Data S3 for reliability. In addition, they estimated (a) the onset time of the displacement (*0* = *never; 1* = *after a while*; *2* = *fairly quickly*)*,* (b) the onset time in minutes, and (c) the frequency of the displacement (*0* = *never; 1* = *once shortly; 2* = *many short times; 3* = *continuously*)*.* At completion of the exit-interview, participants provided a written description of their experiences in two open questions (see ‘testimonies’) and rated 10 statements pertaining to their gaming experiences (2 min), e.g., *‘Did you feel in control of the avatar?’,* rated on an scale from *‘0* = *not at all’* to *‘10* = *yes, I fully did,’* see Table S4.

### Statistical analysis

The study had a partial crossover, mixed factorial design with three repeated measures (i.e., movement conditions ABC) on the dependent variables and one independent grouping factor with two levels (task-order ABC or BAC). The three-stage full-body illusion (A-C) had four dependent variables: (i) Explicit Displacement versus (ii) Implicit Displacement (both with four levels); (iii) Total FBI Interview (three levels); and (iv) Temporal Binding (i.e., estimated time minus real time in seconds, three levels). The Game Exit Interview scores measured Part C in individual items. First, each dependent variable (e.g., ‘*Total FBI Interview*’) was analysed separately in a mixed ANOVA with one between-subjects factor (e.g., ‘*Task Order*’ with two levels) and one within-subjects factor (e.g., ‘*Motion Control*’ with three levels: ABC). If no significant task-order effects were found, the dependent variables could be analysed in a one-way repeated measures ANOVA (or a series of paired samples *t*-tests), comparing the three movement conditions within one group. Correlation analyses were performed between measures.

### Ethics approval

The experiment was performed in line with the principles of the Declaration of Helsinki. Approval was granted by Le Comité d'éthique de la Faculté des Sciences psychologiques et de l'Éducation, Université libre de Bruxelles and the Human Research Ethics Committee of the Queensland University of Technology.

### Consent to participate

Informed consent was obtained from all human research participants.

### Supplementary Information

Supplementary Information.

## Data Availability

The data and materials of the experiment are available at https://osf.io/2c9xz/. The experiment was not preregistered.
