# Supplementary material for: Predicting the bodily self in space and time
Source: Sci Rep. 2024 Jun 27;14:14813. doi: 10.1038/s41598-024-65607-y (PMC11208493; doi:10.1038/s41598-024-65607-y)
Supplement: Supplementary file 1 — Supplementary Information. [file 41598_2024_65607_MOESM1_ESM.pdf]

Supplementary Materials for

Predicting the bodily self in space and time

D.M.L. de Boer[1,2,3]*, P.J. Johnston[3], F. Namdar[4], G. Kerr[1], A. Cleeremans[2].

Correspondence to: demarlou@gmail.com

This file includes:

Table S1-4

Fig. S1-3

Data S1

Data S2

Data S3

Data S4

Other Supplementary Materials for this manuscript include the following:

Data S5 (OSF)

Game Demo (OSF)

Prescreening: VR-safety and Exclusion Criteria

1. Susceptibility: scoring within top 10% of 'time Perceptual Aberration Scale' – tPAS..................(Arzy et al., 2011);
2. Current/history of psychological and neurological disorders;
3. Uncorrected vision/hearing or functional vision problems (e.g. lazy eye, blurred vision, stereoblindness);
4. Balance and/or vestibular problems (incl. motion sickness, problems with 3D-content)...(Birckhead et al., 2019);
5. Current/history of brain-related injury, condition or disease (e.g. epilepsy, stroke, migraine)....(Rossi et al., 2009);
6. Pregnancy..(Rossi et al.);
7. Psychoactive medication, alcohol or recreational drug consumption 24 hours prior to testing...........(Rossi et al.).

Table S1 Prescreening: VR-safety and Exclusion Criteria

Participants were prescreened online and excluded when reporting: (i) high susceptibility to body transfer illusions (Arzy et al., 2011); (ii) current or history of psycho- or neurological disorders; (iii) uncorrected vision, hearing or any functional vision problems; (iv) balance or vestibular problems (e.g., motion sickness, problems with 3D-content (Birckhead et al., 2019); (v) current or history of brain-related injury, condition or disease (e.g., epilepsy, concussion); (vi) pregnancy; or (vii) using psychoactive medication, alcohol or recreational drugs within 24 hours before the experiment (Rossi et al., 2009).

1.	Nausea	Absent/Mild/Moderate/Severe
2.	General Discomfort	Absent/Mild/Moderate/Severe
3.	Stomach Awareness	Absent/Mild/Moderate/Severe
4.	Sweating	Absent/Mild/Moderate/Severe
5.	Increased Salivation	Absent/Mild/Moderate/Severe
6.	Difficulty Concentrating	Absent/Mild/Moderate/Severe
7.	Vertigo	Absent/Mild/Moderate/Severe
8.	Burping	Absent/Mild/Moderate/Severe
9.	Dizziness with Eyes Open	Absent/Mild/Moderate/Severe
10.	Fullness of Head	Absent/Mild/Moderate/Severe
11.	Dizziness with Eyes Closed	Absent/Mild/Moderate/Severe
12.	Difficulty Focusing	Absent/Mild/Moderate/Severe
13.	Headache	Absent/Mild/Moderate/Severe
14.	Blurred Vision	Absent/Mild/Moderate/Severe
15.	Fatigue	Absent/Mild/Moderate/Severe
16.	Eye Strain	Absent/Mild/Moderate/Severe
17.	Anything else?	…………………………………………………

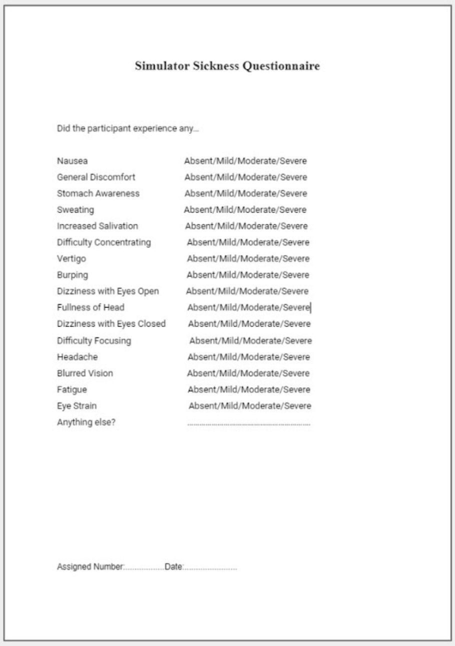
Simulator Sickness Questionnaire

Did the participant experience any…

Nausea	Absent/Mild/Moderate/Severe
General Discomfort	Absent/Mild/Moderate/Severe
Stomach Awareness	Absent/Mild/Moderate/Severe
Sweating	Absent/Mild/Moderate/Severe
Increased Salivation	Absent/Mild/Moderate/Severe
Difficulty Concentrating	Absent/Mild/Moderate/Severe
Vertigo	Absent/Mild/Moderate/Severe
Burping	Absent/Mild/Moderate/Severe
Dizziness with Eyes Open	Absent/Mild/Moderate/Severe
Fullness of Head	Absent/Mild/Moderate/Severe
Dizziness with Eyes Closed	Absent/Mild/Moderate/Severe
Difficulty Focusing	Absent/Mild/Moderate/Severe
Headache	Absent/Mild/Moderate/Severe
Blurred Vision	Absent/Mild/Moderate/Severe
Fatigue	Absent/Mild/Moderate/Severe
Eye Strain	Absent/Mild/Moderate/Severe
Anything else?	…………………………

Assigned Number:……………Date:……………

Table S2 Simulator Sickness Questionnaire

Immediately after completion of the experiment participants were checked for symptoms 1-16 of simulation sickness (e.g., nausea, dizziness, headache). An adapted version of the *Simulator Sickness Questionnaire* was used (Kennedy et al., 1993; Birckhead et al., 2019).

1. (C1) I felt that the body I was looking at was strange.
2. (D1) I felt a shift out of my body towards the virtual body.
3. (C2) I felt as if I had more than one body.
4. (D2) I felt as if I was in front of my body at some point (what I felt came from in between my own and virtual body).
5. (ID1) I felt that the virtual body was my body that was being stroked (or moving in the game).
6. (ID2 reversed) I felt that the body I was looking at was not me.
7. (D3) I felt as if I was behind my body at some point (what I saw and felt happened in front of me).
8. (A1 reversed; D4) I felt a strange or funny feeling at some point (loss of control).
9. (C3) I felt as if I had no body.
10. (ID3 reversed) I felt like someone else's body was in front of me (what I felt and saw was not at the same location).
11. (A2; D5) I felt like I could walk away with the virtual body (as if it was me at that location).
12. (D6 reversed) I felt no shifting towards the virtual body at any point.
13. (D7) I felt my own and virtual body moving closer to each other.
14. (A3; D8 reversed) I felt that I had control over the virtual body.
15. (ID4) I felt that the body I was looking at was me.

2. I felt a shift out of my body towards the virtual body.

	Strongly agree	Agree	Neither agree nor disagree	Disagree	Strongly disagree
Body illusion	☐	☐	☐	☐	☐
Self-stroking	☐	☐	☐	☐	☐
Gaming	☐	☐	☐	☐	☐

Table S3 15-item FBI Exit Interview

Participants indicated their agreement to three sets (Part A 'Body illusion', Part B 'Self-stroking', Part C 'Gaming' below right) of 15 statements, answered on 5-point Likert scales ranging from *'1 = Strongly Disagree'* to *'5 = Strongly Agree.'* The statements measured altered self-location or *'Displacement'* (D1-8), *'Self-Identification'* (i.e., body-ownership; ID1-4) and *'Sense of Agency'* (A1-3). C1-3 were control items; C1 also measured participants' disposition towards the virtual body in Part 1-3.

Table S4. Game Exit-interview

1. Did you feel in control of the avatar? *“Not at all” 0 - 1 - 2 - 3 - 4 - 5 - 6 - 7 - 8 - 9 -10 “Yes, I fully did”*
2. Did you feel a connection with the avatar (as an extension of you)? *0 - 1 - 2 - 3 - 4 - 5 - 6 - 7 - 8 - 9 -10*
3. Could you still distinguish yourself from the avatar? *0 - 1 - 2 - 3 - 4 - 5 - 6 - 7 - 8 - 9 -10*
4. Did you feel immersed into the game (as if the avatar was you)? *0 - 1 - 2 - 3 - 4 - 5 - 6 - 7 - 8 - 9 -10*
5. Did you feel displaced towards the avatar at any point (as if being at that location)? *0 - 1 - 2 - 3 - 4 - 5 - 6 - 7 - 8 - 9 -10*
6. Did it feel uncomfortable when your avatar got hit (as if it might be you)? *0 - 1 - 2 - 3 - 4 - 5 - 6 - 7 - 8 - 9 -10*
7. Did time pass by quickly whilst you were playing? *0 - 1 - 2 - 3 - 4 - 5 - 6 - 7 - 8 - 9 -10*
8. Did you experience a flow in your performance? *0 - 1 - 2 - 3 - 4 - 5 - 6 - 7 - 8 - 9 -10*
9. Were you satisfied with your performance? *0 - 1 - 2 - 3 - 4 - 5 - 6 - 7 - 8 - 9 -10*
10. Was the game fun to play (would you play it again)? *0 - 1 - 2 - 3 - 4 - 5 - 6 - 7 - 8 - 9 -10*

Table S4 10-item Game Exit Interview

Participants rated their gaming experiences on 10 items, answered on 11-point rating scales ranging from *‘0 = not at all’* to *‘10 = yes, I fully did.’* In addition to gaming-related aspects (e.g., flow, immersion, satisfaction), it also measured shifts in self-location, self-identification and sense of agency.

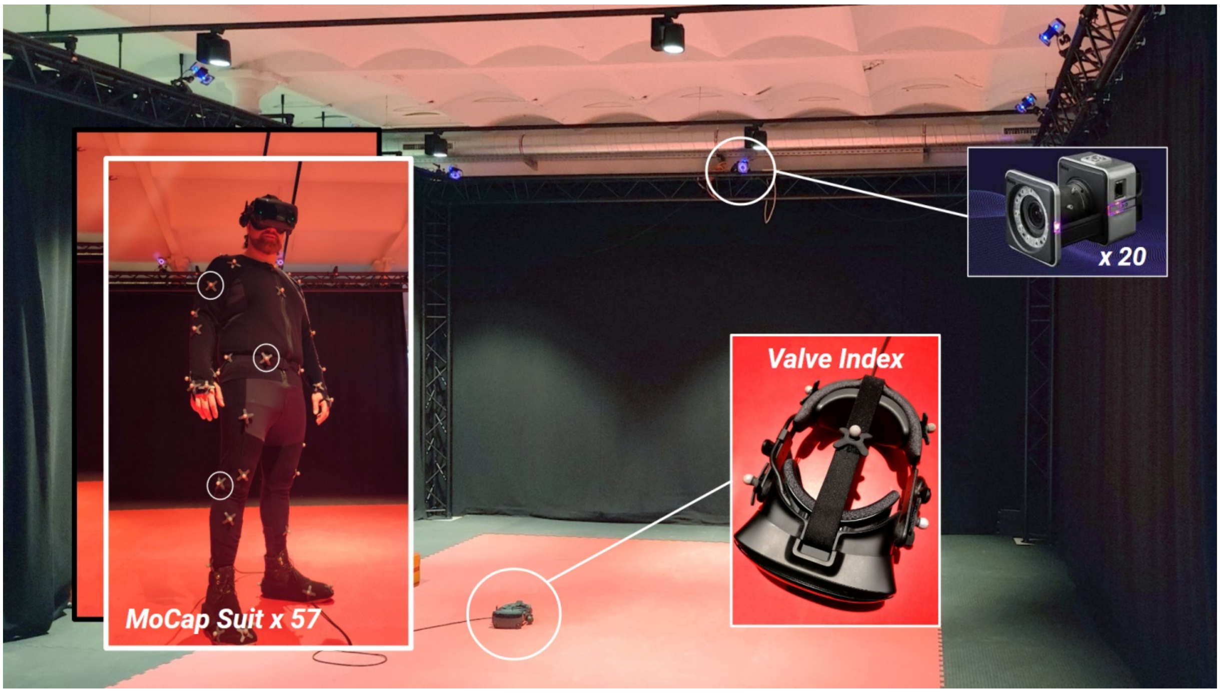

Figure S1 Index-VR combined with Vicon Motion Capture

Laboratory space (11 x 7 metres) and materials used to combine VR (Valve Index) with real-time motion capture (Vicon). Vicon systems rely on 'passive optical motion capture' using retroreflective markers that are strategically placed onto bodies (left) or objects (right, Valve Index) to best recover their original shape and/or body pose. Participants wore a MoCap Suit with 52 markers and VR-headset with five markers (total = 57). Our setup used 20 Vero cameras v2.2 (above right) to track the position of the 57 markers with up to 0.017 mm dynamic accuracy.

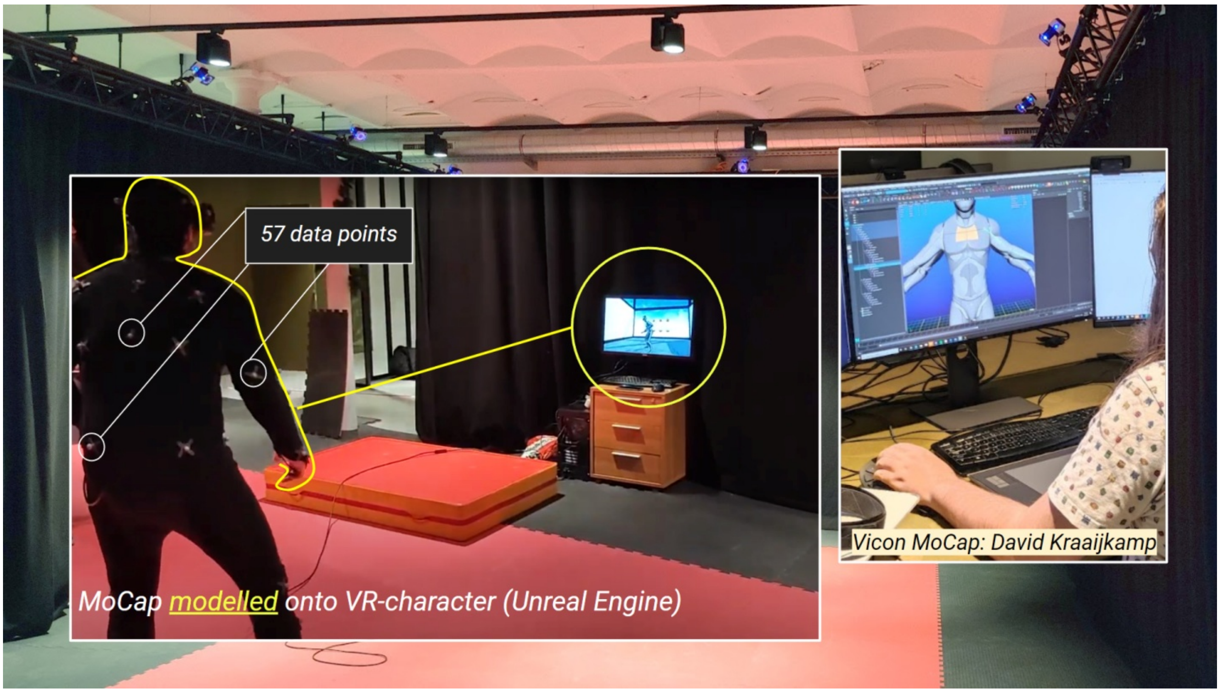

Figure S2 MoCap modelled onto 3D Unreal humanoid

Motion information LIVE-captured from the participant was used by Unreal Engine 4.26.2 to real time model movements onto a full-sized 3D humanoid standing in front of the participant. Live Link Plugin 1.5 for Unreal Engine 4 allowed the Vicon system (PC 1) to real-time stream the data of 57 captured points to the Skeletal Mesh of a basic character in Unreal (PC 2; VR setup).

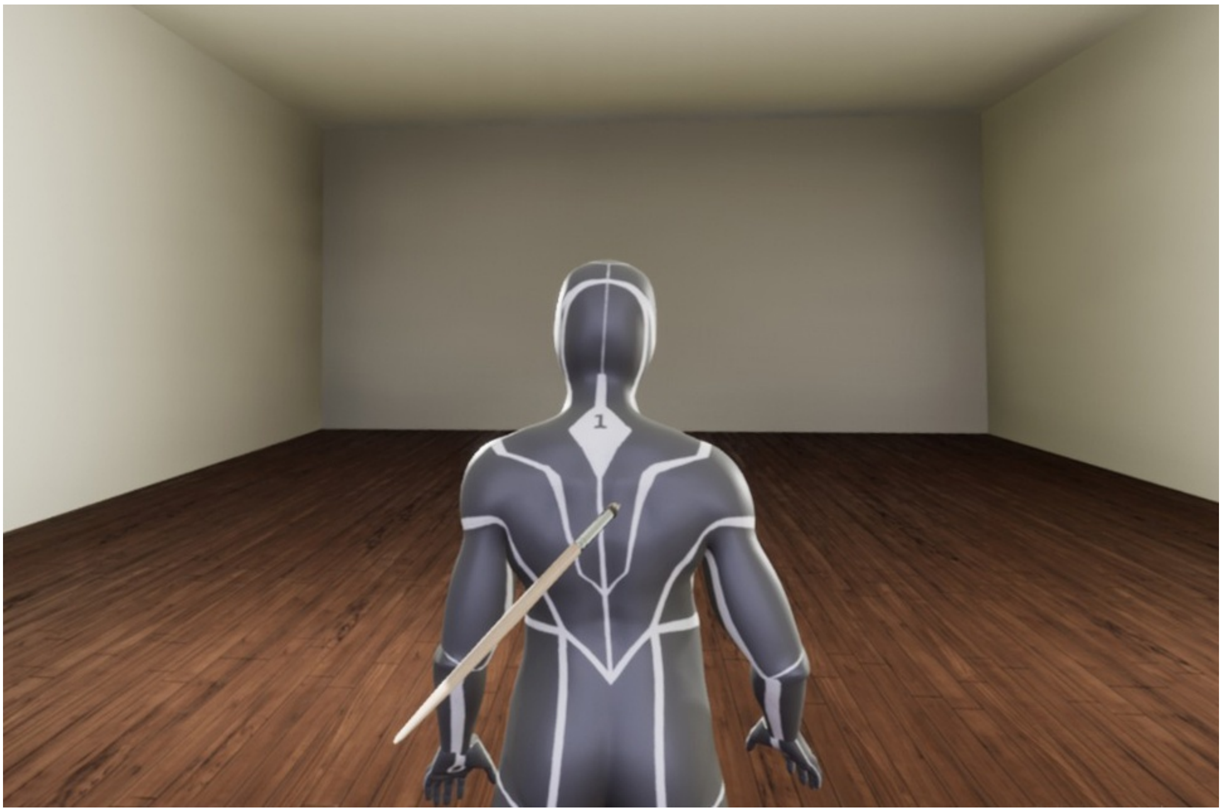

Figure S3 ‘Empty Room’ simulation in Unreal

Unreal Engine 4.26.2 was used to create and real-time render two simulations in VR: (i) one basic ‘Empty Room’ simulation for the Full-body Illusions (Part A & B; depicted above); and (ii) a similarly shaped ‘Game Arena’ for the Immersive Game (Part C; see Fig. S2). Here the passive illusion (Part A) is shown with the 3D humanoid wearing a Vicon suit (development stage) and 3D modelled brush.

Data S1. Results Game Exit Interview

The Game Exit Interview consisted of 10 items, answered on an 11-point rating scale ranging from *‘0 = not at all’* to *‘10 = yes, I fully did’* (n = 40). The Game was perceived as extremely fun to play (Item 10; M = 9), high in self-control (Item 1; sense of agency; M = 8.45), losing sense of time (Item 7; Intentional Binding; M = 8.23), high in self-identification / low in dissociation (Item 3; self-identification; M = 1.75), highly engaging (Item 2; self-identification; M = 7.68), highly immersive (Item 4; self-identification; M = 7.65), strong in flow (Item 8; perceived control over time; M = 7.55), grounded (Item 5; Displacement low; M = 3.48), rewarding (Item 9; M = 6.9) and low in negative affect (Item 6; M = 4.88).

Data S2. Temporal Binding

The 'temporal binding' measure is an implicit measure (or index) of how 'salient' a phenomenal experience is: salient sensory information / events are grouped together in the brain, subjectively shortening perceived time (Venskus et al., 2021). Thus, (i) in case a true shift in self-location occurred (i.e., a salient sensory event) we expected this to correlate with people's estimated time (i.e., the time passed is estimated as shorter than the real time passed); whereas (ii) when no shift in self-location was reported, the time passed should be estimated as closer to the real time passed (or longer in case of boredom). Note: this measure should not be mistaken for 'intentional binding' that concerns the subjective shortening of time when actions are voluntary. Passive illusions cannot be measured with intentional binding because there is no self-produced signal (i.e., intention) to 'bind' events in time (Haggard, Clark, & Kalogeras, 2002).

Calculations:
Part 1 / passive illusion: estimated time minus real time in seconds;
Part 2 / active illusion: estimated time minus real time in seconds;
Part 3 / immersive game: estimated play time minus real play time in seconds.

Data S3. Reliability FBI Exit Interview

Negatively phrased questions of the 15-item FBI exit interview were reverse coded, and did not indicate inconsistencies or recklessness in answering. Descriptive statistics confirmed that all items had a mean score around 3 points and SD > 1 point (Part 1: except control item 9 & item 14; Part 3: except control items 1, 9 & item 14; Range 1-5 points on a Likert scale). As expected, the displacement items (Items 2, 4, 7, 8, 12, 13 & 14) were strongly correlated, $r(39) > .5, p = .01$; whilst the other items including item 11 'high Agency' were not correlated, $r = 0$ one-tailed Bonferroni corrected. Cronbach's α: .86 Part A; .84 Part C (displacement items); .85 Part A; .84 Part C (all items except control items 1, 3 & 9). Of note, the virtual body was perceived as less strange in the game ($M = 2.1$) than in the illusions (Part 1 $M = 2.9$, Part 2 $M = 3$; Item 1, FBI Disposition).

Data S4. Technical Requirements Methods & Limitations

The Valve Index system minimally requires a gaming PC with 8GB of RAM, an NVIDIA GeForce GTX 970 or AMD RX480 graphics card and a dual-core CPU with Hyper-Threading (note: to translate and real-time render the motion capture data to simulations in Unreal we required an AMD 5800x supercomputer with 128GB of RAM, and NVIDIA GeForce RTX 3080 Ti graphics card).

The Vicon system requirements of up to 40 cameras are: a Precision 3650 Tower (XE) or equivalent; CPU: 11th Generation Intel® Core™ i7-11700 (8-core, 16 MB cache, base 2.5 GHz, up to 4.9 GHz); Graphics card: NVIDIA® GeForce RTX™ 3060 / NVIDIA RTX A4000; RAM: 16 GB (2X8 GB) DDR4 UDIMM non-ECC; OS Hard Drive: 512 GB PCIe NVMe Class 40 M.2 SSD; Video Hard Drive: 1 TB PCIe NVMe CLASS 40 M.2 SSD; Data Hard Drive: 2 TB 7200 rpm SATA 3.5" HDD; Network Card: Intel Ethernet 10G Dual Port X550-T Adapter (2X10 GbE) NIC PCIe Card; Wireless Adapter: Intel Wi-Fi 6 AX201 802.11ax Dual Band (2×2) Wireless module + Bluetooth® 5.2. Note on the specified latency: at 1000 Hz the latency on the camera + transmission to the PC should be in the region of 1.5 ms for commonly used marker sets between 10 and 40 markers. However, the processing that takes place on the PC will add 1-5 ms depending upon the specifics of the PC, the number of markers/cameras, the complexity of the labelling problem and other processes/services that compete for the CPU time.

Several technical- and anatomical limitations prevented our participants from stroking themselves on the back. This is difficult even without wearing a motion capture suit with motion trackers attached to it. Stroking applied to the lower back with the back of the hand caused the motion trackers to rub against each other, come off and/or get displaced. Similarly, indirect methods using tools appeared to seriously interfere with the motion tracking (i.e., distorting the movements and physical appearance of the virtual body). A previously tried alternative (using video recordings) was 'neck-stroking' (Swinkels et al., 2021). However, because most of our participants showed insufficient overlap in the hand- and face-models of their avatar this made the stroking look unreal (e.g., the hand and neck would not visibly touch). Again, adding more markers to our setup would cause too much interference in the data stream and the software to crash (see *Method*). 'Belly-stroking,' however, appeared to be a feasible option that worked well for every body type. Although this difference in visuotactile stimulation potentially confounded the comparison between the conditions, it appeared that belly- versus neck-stroking in five individuals showing sufficient virtual hand-face overlap gave identical results: no shifts in self-location were reported (whilst three of them did report this in the passive illusion).

Data S5. (OSF) https://osf.io/2c9xz/

Game Demo (OSF) https://osf.io/yfxgv
